# β-Receptor blocker enhances the anabolic effect of PTH after osteoporotic fracture

**DOI:** 10.1038/s41413-024-00321-z

**Published:** 2024-03-21

**Authors:** Jie Huang, Tong Wu, Yi-Rong Jiang, Xuan-Qi Zheng, Huan Wang, Hao Liu, Hong Wang, Hui-Jie Leng, Dong-Wei Fan, Wan-Qiong Yuan, Chun-Li Song

**Affiliations:** 1 Department of Orthopedics, Peking University Third Hospital, 100191 Beijing, China; 2 Beijing Key Laboratory of Spinal Disease, 100191 Beijing, China; 3 Engineering Research Center of Bone and Joint Precision Medicine, 100191 Beijing, China

**Keywords:** Osteoporosis, Osteoporosis

## Abstract

The autonomic nervous system plays a crucial role in regulating bone metabolism, with sympathetic activation stimulating bone resorption and inhibiting bone formation. We found that fractures lead to increased sympathetic tone, enhanced osteoclast resorption, decreased osteoblast formation, and thus hastened systemic bone loss in ovariectomized (OVX) mice. However, the combined administration of parathyroid hormone (PTH) and the β-receptor blocker propranolol dramatically promoted systemic bone formation and osteoporotic fracture healing in OVX mice. The effect of this treatment is superior to that of treatment with PTH or propranolol alone. In vitro, the sympathetic neurotransmitter norepinephrine (NE) suppressed PTH-induced osteoblast differentiation and mineralization, which was rescued by propranolol. Moreover, NE decreased the PTH-induced expression of Runx2 but enhanced the expression of *Rankl* and the effect of PTH-stimulated osteoblasts on osteoclastic differentiation, whereas these effects were reversed by propranolol. Furthermore, PTH increased the expression of the circadian clock gene *Bmal1*, which was inhibited by NE-βAR signaling. *Bmal1* knockdown blocked the rescue effect of propranolol on the NE-induced decrease in PTH-stimulated osteoblast differentiation. Taken together, these results suggest that propranolol enhances the anabolic effect of PTH in preventing systemic bone loss following osteoporotic fracture by blocking the negative effects of sympathetic signaling on PTH anabolism.

## Introduction

Osteoporotic fractures are becoming more prevalent as the population ages.^1^ Patients with osteoporosis-related fractures not only encounter difficulties in fracture healing but also have an elevated risk of subsequent fractures, which is a challenging issue in clinical practice.^2,3^ Previous studies have indicated that fracture induces acute systemic bone loss, increasing the risk of subsequent fractures.^4–6^ However, the underlying mechanism remains unclear, and few studies have investigated therapeutic strategies for preventing systemic bone loss and fracture recurrence following fractures.

The use of anti-osteoporosis drugs after fractures is controversial due to their impact on bone remodeling, which is essential for fracture healing.^7^ Teriparatide, recombinant human parathyroid hormone (rhPTH1-34), is the most commonly used anabolic agent in the clinic. Intermittent PTH administration strongly promotes bone formation and reduces the risk of fractures.^2^ Despite not being approved for fracture treatment, preclinical and clinical studies have shown promising therapeutic effects of PTH on fracture healing.^8,9^

Bone metabolism is regulated by a complex interplay of various signaling pathways and cellular processes. One of the major regulators of bone metabolism is the sympathetic nervous system, which suppresses bone formation via osteoblast β-adrenergic receptor (βAR) signaling.^10,11^ Propranolol, a nonselective βAR antagonist, ameliorated hypertension-induced or depression-induced bone loss.^12,13^ Interestingly, there is a functional interaction between parathyroid hormone 1 receptor (PTH1R) and β2AR signaling, with β2AR deficiency hindering both PTH-induced increases in bone formation and resorption.^14^ These findings indicate that the two signaling pathways are connected and may interact in the maintenance of bone metabolism. Given the sympathetic stress induced after fracture, we propose that combination of PTH and propranolol may contribute to a better therapeutic effect on systemic bone loss after osteoporotic fracture.

Bone metabolism exhibits circadian rhythms, represented by the diurnal fluctuation of bone turnover markers and bone metabolism-regulating factors, including PTH.^15^ It is well-established that circadian clock genes play an important role in regulating bone metabolism, and knockout of these genes results in significant changes in the bone mass phenotype.^15,16^ Teriparatide administration at different times of day (morning or evening) results in significant changes in the 24-h variation in bone turnover markers in postmenopausal osteoporotic women.^17^ Additionally, βAR signaling in osteoblasts promotes osteoclastogenesis but also restrains bone formation by inhibiting osteoblast proliferation via a Clock-dependent mechanism.^10,18^ These findings motivated us to investigate whether circadian clock genes are involved in bone remodeling stimulated by the combination therapy comprising PTH and propranolol.

In this study, we report that propranolol augments the positive effect of PTH on systemic bone formation and fracture healing in mice with osteoporotic fractures. Furthermore, we demonstrated that the activation of βAR signaling by NE represses PTH-induced osteogenesis and augments the effect of PTH-induced osteoblasts on osteoclast formation, which is reversed by propranolol. Mechanistically, NE-βAR signaling inhibits PTH-induced RUNX2 expression and osteoblast differentiation by regulating BMAL1. In summary, our study elucidates the ability of propranolol to enhance the anabolic effect of PTH on systemic bone loss after osteoporotic fracture and the underlying mechanism, which may provide a novel therapeutic strategy for the treatment of systemic bone loss after osteoporotic fracture.

## Results

### Propranolol improves the bone anabolic effect of PTH after osteoporotic fracture

**Fig. 1:**
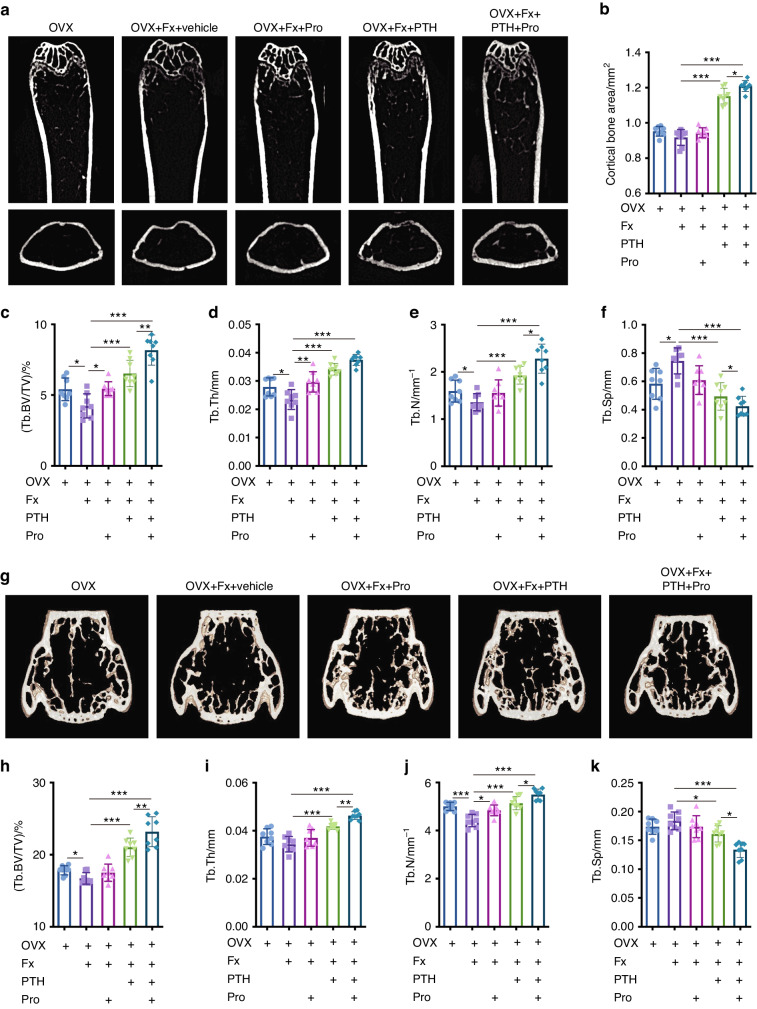
Propranolol increases PTH-induced bone formation in femurs and L5 in OVX mice after fracture. **a** Representative μCT images of femurs from OVX, OVX + Fx (fracture), and OVX + Fx mice treated with Pro (propranolol), PTH or PTH + Pro. Quantitative μCT analysis of the cortical bone area (**b**), trabecular bone volume fraction (Tb. BV/TV; **c**), trabecular bone thickness (Tb. Th; **d**), trabecular bone number (Tb. N; **e**) trabecular bone separation (Tb. Sp; **f**); *n* = 8. **g** Representative μCT images of L5. **h**–**k** Quantitative μCT analysis of trabecular bone microarchitecture of L5; *n* = 8. The data are presented as the mean ± SD. An unpaired, two-tailed Student’s *t* test was used to test the differences between the OVX and OVX + Fx groups; two-way ANOVA combined with Tukey’s post hoc test was used to test the differences between the OVX + Fx and OVX + Fx mice treated with Pro, PTH or PTH + Pro. *P < 0.05, **P < 0.01, ***P < 0.001

To examine the effects of PTH and propranolol on systemic bone loss after osteoporotic fracture, we generated tibia fracture models in mice with ovariectomy (OVX)-induced postmenopausal osteoporosis. The mice were administered PTH, propranolol, PTH + propranolol or vehicle for 4 weeks after fracture. μCT analysis of femurs revealed that fracture resulted in significantly decreased trabecular bone mass in OVX mice, which was determined by quantitative analyses of trabecular bone volume fraction (Tb. BV/TV), trabecular thickness (Tb. Th), number (Tb. N) and separation (Tb. Sp) (Fig. 1a–f). Propranolol administration mildly but significantly increased the trabecular bone volume fraction and trabecular thickness (Fig. 1a, c, d). Moreover, compared with vehicle-treated mice, PTH-treated mice had dramatically increased cortical bone and trabecular bone volumes (Fig. 1a–f). Importantly, the combined treatment with PTH and propranolol increased cortical bone and trabecular bone mass to a greater extent than treatment with either agent alone (Fig. 1a–f). Specifically, compared to the vehicle group, the trabecular bone volume fraction was 54% greater in mice treated with PTH alone, and was 93% greater in mice treated with the combination of PTH and propranolol. Similar results were observed in the μCT analysis of the fifth lumbar vertebra (L5). Fracture also led to a significant decrease in the bone volume at L5 (Fig. 1g–k). However, the mice treated with PTH showed a 26% increase in the trabecular bone volume fraction of L5, and the mice treated with both PTH and propranolol exhibited a 47% greater trabecular bone volume fraction than did the vehicle group (Fig. 1g–k), indicating that propranolol increased the osteogenic effect of PTH by approximately 80% in mice with osteoporotic fractures.

We further evaluated the combined effects of PTH and propranolol on normal mice after fracture. Tibial fractures resulted in significant systemic bone loss in mice, as evidenced by the significantly decreased trabecular bone volume in femurs and L5 (Fig. S1a–k). However, propranolol treatment reduced systemic bone loss after fracture and PTH administration dramatically improved the bone mass and microstructure in the femur and L5 following fracture (Fig. S1a–k). Notably, the combined use of propranolol further augmented the anabolic effect of PTH.

These findings confirm the occurrence of fracture-induced systemic bone loss in mice with or without OVX and the greater efficacy of the combination of PTH and propranolol than of PTH or propranolol alone in treating systemic bone loss after fracture.

### Propranolol enhances osteoblastic bone formation and suppresses osteoclastic bone resorption in PTH-treated mice with osteoporotic fractures

**Fig. 2:**
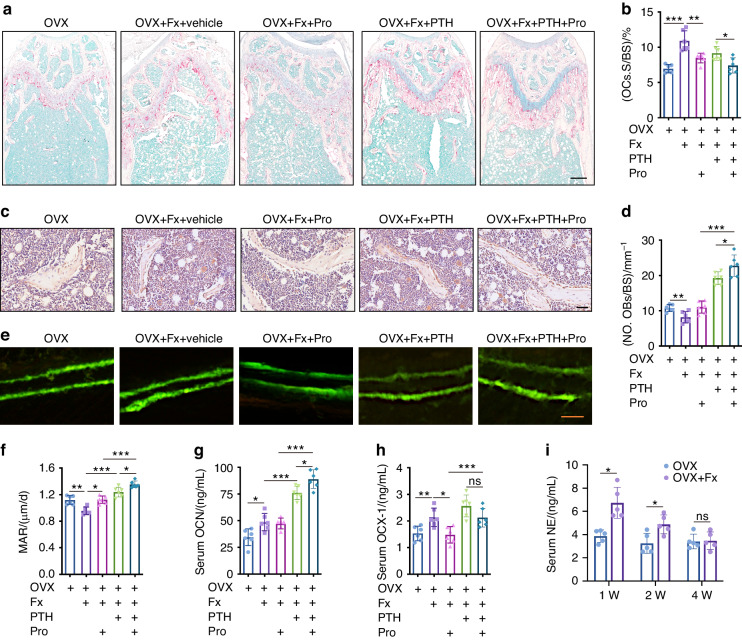
Propranolol increases osteoblast bone formation and suppresses osteoclast resorption in PTH-treated OVX mice after fracture. **a** TRAP staining of femurs from the different groups as indicated. Scale bar: 250 μm. **b** Quantification of the TRAP^+^ osteoclast surface (OCs. S) per bone surface (BS) in the different treatment groups. *n* = 6 per group. **c** Representative images of OCN immunostaining in distal femurs from different groups as indicated. Scale bar: 50 μm. **d** Quantification of the number of OCN-stained osteoblasts (No. OBs) on trabecular BS in distal femurs. *n* = 6 per group. Representative calcein double labeling of distal femurs from different groups (**e**) and quantification of the mineral apposition rate (MAR) (**f**). Scale bar: 10 μm. *n* = 6 per group. **g**, **h** ELISA analysis of the serum concentrations of OCN and CTX-I. **i** ELISA analysis of the serum concentration of NE at week 1, 2 and 4 in OVX mice with or without fracture. *n* = 5 per group. The data are presented as the mean ± SD. For panels **b**, **d**, **f**–**h**: an unpaired, two-tailed Student’s *t* test was used to test the differences between OVX and OVX + Fx groups; two-way ANOVA combined with Tukey’s post hoc test was used to test the differences between OVX + Fx, OVX + Fx mice treated with Pro, PTH or PTH + Pro groups. For panel **i**: two-way ANOVA combined with the Bonferroni post hoc correction was used to test the differences between the OVX and OVX + Fx groups at different time points. *P < 0.05, **P < 0.01, ***P < 0.001

TRAP (tartrate-resistant acid phosphatase) staining revealed that fractures induced markedly more osteoclast formation on the bone surface of femurs in the OVX mice than in the control mice (Fig. 2a, b). However, both PTH and propranolol treatment reduced osteoclast formation in the femurs of OVX mice after fracture, and the combination of PTH and propranolol inhibited osteoclast formation to a greater extent than did PTH alone (Fig. 2a, b). Immunohistochemical staining for osteocalcin (OCN) showed that the number of OCN^+^ osteoblast on the trabecular bone surface was significantly reduced in OVX mice after fracture but increased when the mice were treated with PTH (Fig. 2c, d). Compared to that of PTH or propranolol alone, the cotreatment of PTH and propranolol resulted in significantly increased osteoblast formation in OVX mice after fracture (Fig. 2c, d). Calcine double labeling further confirmed the fracture-induced decrease in the mineral apposition rate (MAR) in OVX mice, which was significantly improved by both propranolol and PTH treatment and peaked under PTH + propranolol intervention (Fig. 2e, f). An increase in the serum OCN concentration and a decrease in the serum levels of the resorption marker C-terminal telopeptide of type I collagen (CTX-I) were detected via enzyme-linked immunosorbent assay (ELISA) in the mice cotreated with PTH and propranolol relative to the mice treated with PTH alone (Fig. 2g, h). Sympathetic tone, determined by the serum norepinephrine (NE) concentration, was significantly increased 2 weeks after fracture and returned to normal by 4 weeks after fracture (Fig. 2i). These findings suggest that propranolol enhances the anabolic effect and suppresses the catabolic effect of PTH, which may be attributed to propranolol’s competitive inhibition of the βAR agonist NE.

### Propranolol facilitates the effect of PTH on osteoporotic fracture healing

**Fig. 3:**
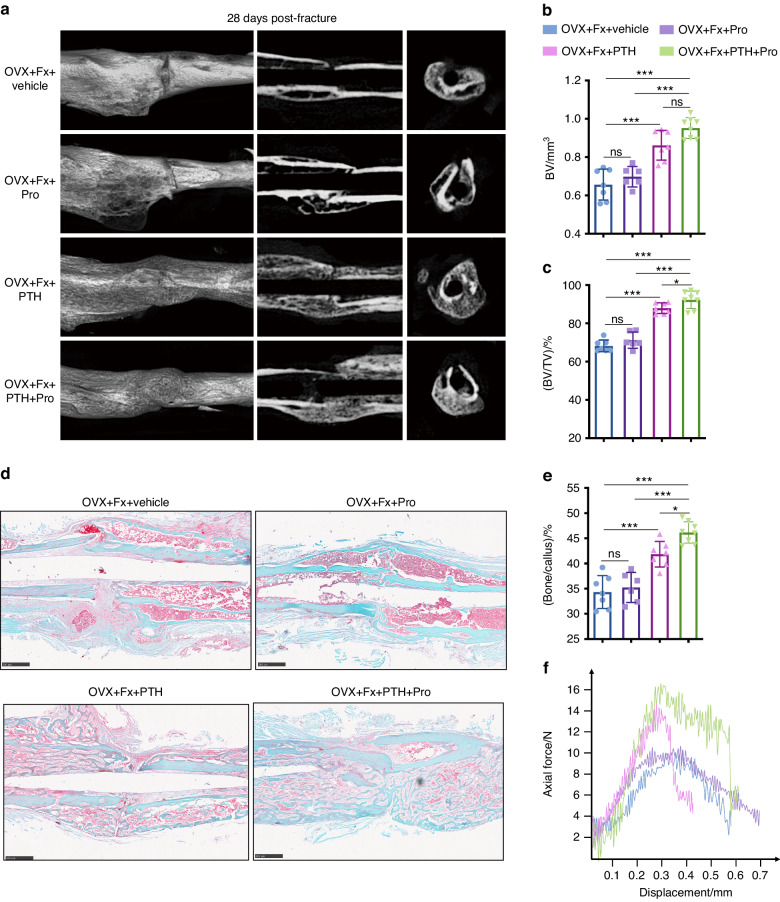
Propranolol facilitates the effect of PTH on osteoporotic fracture healing. **a** μCT 3D reconstructions and axial cross-sectional and coronal cross-sectional images of fractured tibias from OVX mice treated with PTH, Pro, PTH + Pro or vehicle on day 28 after fracture. **b**, **c** Quantitative analysis of the bone volume (BV) and the bone volume fraction (BV/TV) of the calluses. *n* = 6–8 per group. **d** Safranin-O/fast green staining images of fractured tibia sections from the different groups as indicated. Scale bar: 500 μm. **e** Quantitative analysis of the bone fraction in the calluses of the different groups. *n* = 6–8 per group. **f** Three-point bending measurement of the ultimate load on fractured tibias from different groups. The data are presented as the mean ± SD. Two-way ANOVA combined with the Tukey’s post hoc test was used to test the differences among all groups. *P < 0.05, **P < 0.01, ***P < 0.001

We further assessed the impact of PTH and propranolol on fracture healing in OVX mice via μCT scanning 28 days after fracture. As shown in Fig. 3a, OVX mice exhibited impaired fracture healing, characterized by little callus formation and a lack of bridging callus (Fig. 3a–c). No significant differences were observed in the bone volume (BV) or BV fraction (BV/TV) of the calluses between the propranolol and vehicle groups (Fig. 3a–c). However, mice administered PTH showed significantly increased BV and BV/TV in calluses compared to the vehicle group, whereas higher BV and BV/TV in calluses were observed in mice treated with PTH + propranolol than in those treated with propranolol or PTH alone (Fig. 3a–c). Similar fracture healing results were observed in fractured mice without OVX administrated with PTH and propranolol (Fig. S2a–c). We also detected the NE levels in the fracture sites and serum of the sham and OVX mice, and there was no significant difference in both the fracture sites and serum between the two groups (Fig. S3a–d).

Safranin-O/fast green staining was further performed to examine hard callus formation in the fracture site. Consistent with the results of the μCT analyses, mice treated with PTH had markedly greater hard callus formation than that of the vehicle group, and the combination of PTH and propranolol induced a significantly greater hard callus fraction compared to mice treated with propranolol or PTH alone (Fig. 3d, e). The hard callus fraction was not significantly different between the propranolol and vehicle treatment groups (Fig. 3d, e). A three-point bending test was performed to evaluate the biomechanical properties of the fractured tibia. The bone strength of the fractured tibia in mice treated with propranolol was similar to that in the vehicle group, but PTH treatment resulted in a marked increase in the maximum loading force relative to that in the vehicle group (Fig. 3f). However, the mice administered PTH + propranolol had the highest maximum loading force among all the groups.

Considering the adverse effects of propranolol, such as fatigue and insomnia,^19,20^ we monitored the body weight of the mice in each group, detected liver and kidney function, and examined fatigue levels using rotarod tests. No significant differences were found in the body weight (Fig. S4a); liver and kidney function markers levels, including aspartate transaminase (AST), alanine transaminase (ALP), creatinine (Cr), and blood urea nitrogen (BUN), among the groups (Fig. S4c–f). On day 28 post-fracture, the fractures led to a notable decrease in latency time in the rotarod tests. However, no statistically significant differences were observed among the fractured mice treated with propranolol, PTH, PTH + propranolol, or vehicle treatment (Fig. S4g). These findings suggest that the administration of PTH and propranolol for a duration of 4 weeks does not induce evident adverse effects in mice with fractures.

Taken together, these findings suggest that PTH facilitates fracture healing and that propranolol enhances the effect of PTH on promoting fracture healing.

### Activation of βAR signaling suppresses PTH-induced osteoblast differentiation

**Fig. 4:**
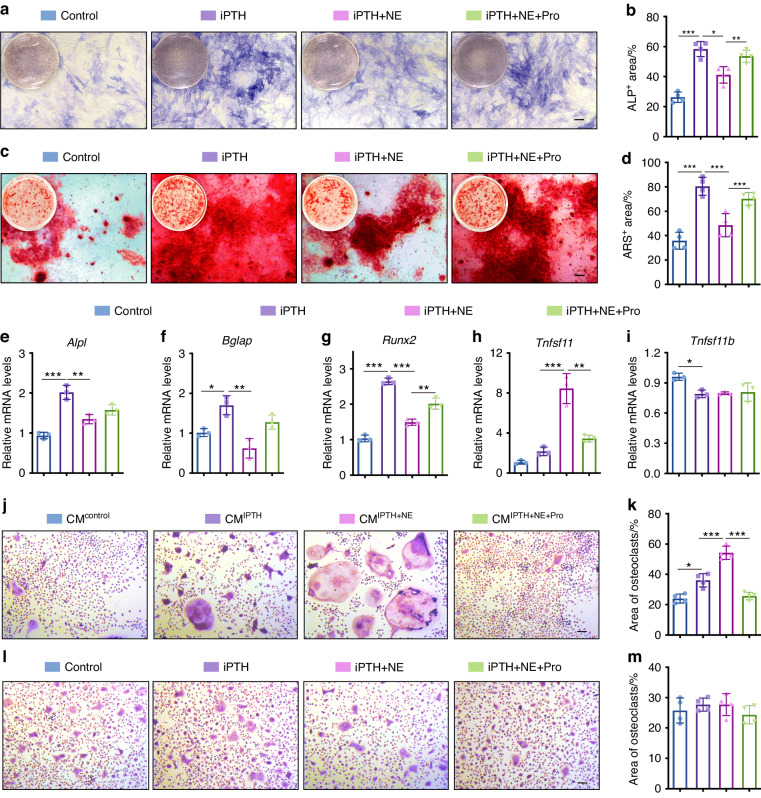
NE suppresses the positive effect of iPTH on osteoblast differentiation, but enhances the ability of PTH-induced osteoblasts to promote osteoclastic differentiation. **a** Alkaline phosphatase (ALP) staining images of BMSCs treated with vehicle (control), iPTH, iPTH + NE or iPTH + NE + propranolol under osteogenic induction. Scale bar: 50 μm. **b** Quantification of the ALP positive areas. *n* = 5 per group. **c** Alizarin red staining (ARS) images of the different groups under osteogenic induction. Scale bar: 50 μm. **d** Quantification of the ARS positive areas. *n* = 5 per group. **e**–**i** qRT‒PCR analysis of *Alp*, *Osteocalcin*, *Runx2*, *Rankl* and *Opg* mRNA expression in BMSCs receiving different treatments under osteogenic induction for 3 days. *n* = 3 per group. TRAP staining images (**j**) and quantification of TRAP^+^ osteoclasts areas (**k**) in of BMMs treated with CM from different groups under osteoclastogenic induction. Scale bar: 50 μm. *n* = 4 per group. TRAP staining images (**l**) and quantification of TRAP^+^ osteoclast areas (**m**) of BMMs treated with vehicle, iPTH, iPTH + NE or iPTH + NE + propranolol under osteoclastogenic induction. Scale bar: 50 μm. *n* = 4 per group. The data are presented as the mean ± SD. Two-way ANOVA combined with Tukey’s post hoc test was used to test the differences among all groups. *P < 0.05, **P < 0.01, ***P < 0.001

To investigate the potential mechanism by which the combination of PTH and propranolol induces a stronger osteogenic effect in mice after osteoporotic fractures, bone marrow stem cells (BMSCs) were cultured with intermittent PTH (iPTH), iPTH + norepinephrine (NE), iPTH + NE + propranolol or vehicle under osteogenic induction in vitro. Alkaline phosphatase (ALP) and Alizarin Red staining (ARS) revealed that, compared with of the control group, iPTH administration significantly enhanced osteoblast differentiation and calcium nodule formation (Fig. 4a–d). However, NE inhibited the osteogenic effect of iPTH, which was reversed by propranolol intervention (Fig. 4a–d). Similar results were also observed in the mRNA levels of *Alpl* and *Bglap* (osteocalcin), genes expressed during osteogenic differentiation, in BMSCs treated with iPTH, iPTH + NE or iPTH + NE + propranolol (Fig. 4e, f). *Runx2*, an essential transcription factor for osteoblast differentiation, was also enhanced by iPTH treatment, whereas this increase was reversed by combination with NE, which was further reversed by propranolol administration (Fig. 4g). These results suggest that NE inhibits the effect of iPTH on bone differentiation through the βAR signaling pathway, which can be rescued by the β-blocker propranolol.

### Activation of βAR signaling enhances the osteoclastogenesis ability of PTH-stimulated osteoblasts

Osteoblasts govern osteoclast differentiation by secreting receptor activator for nuclear factor-κB ligand (RANKL) and osteoprotegerin (OPG).^21^ Quantitative real-time PCR (qRT‒PCR) analyses determined significantly higher *Tnfsf11* (Rankl) but lower *Tnfsf11b* (OPG) mRNA levels in BMSCs treated with iPTH than in the control, and NE further enhanced the effect of iPTH on *Tnfsf11* expression, which was attenuated by propranolol administration (Fig. 4h, i). To test the effects of iPTH, NE and propranolol on osteoblast-induced osteoclast formation, bone marrow macrophages (BMMs) were isolated and incubated with conditioned media (CM) from BMSCs stimulated with iPTH, iPTH + NE or iPTH + NE + propranolol under osteoclastogenic induction. As shown in Fig. 4j, BMMs treated with CM from iPTH-stimulated BMSCs formed more TRAP^+^ osteoclasts than those treated with CM from control cells, and these effects were further augmented in the group treated with CM from iPTH + NE-treated BMSCs. However, once the iPTH + NE-stimulated cells were additionally treated with propranolol, the pro-osteoclastogenesis effect was inhibited (Fig. 4j, k). We also assessed the direct effects of iPTH, iPTH + NE and iPTH + NE + propranolol on osteoclastogenesis to exclude the possible impact of residual PTH, NE and propranolol in CM on osteoclast differentiation. TRAP^+^ osteoclast quantification showed no obvious differences among the groups directly treated with iPTH, iPTH + NE, iPTH + NE + propranolol or vehicle (Fig. 4l, m), suggesting that the positive effects of iPTH and iPTH + NE on osteoclastogenesis are mediated by osteoblasts. These findings suggest that NE-activated βAR signaling enhances the ability of PTH-stimulated osteoblasts to stimulate osteoclastic differentiation, while propranolol blocks these effects.

### NE inhibits iPTH-induced Bmal1 expression in osteoblasts via βAR signaling

**Fig. 5:**
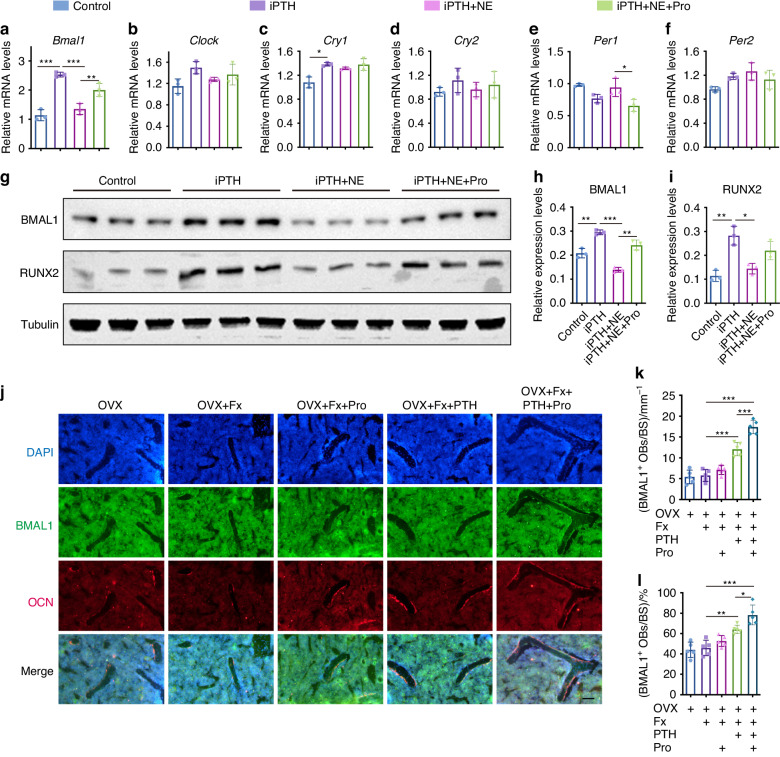
NE inhibits iPTH-induced Bmal1 expression in osteoblasts via βAR signaling. **a**–**f** qRT‒PCR analyses of *Bmal1*, *Clock*, *Cry1*, *Cry2, Pre1* and *Pre2* mRNA expression in BMSCs treated with vehicle, iPTH, iPTH + NE or iPTH + NE + propranolol under osteogenic induction for 3 days. Western blot images (**g**) and quantification of BMAL1 (**h**) and RUNX2 (**i**) relative to tubulin in BMSCs receiving different treatments. *n* = 3 per group. **j** Representative images of co-localization of Bmal1 (green) and OCN (red) immunofluorescent staining in distal femurs from OVX, OVX + Fx, and OVX + Fx mice treated with Pro, PTH or PTH + Pro. Scale bar: 50 μm. **k** Quantification of the number of BMAL1^+^ osteoblasts (OCN^+^) on trabecular BS. *n* = 5 per group. **l** Quantification of the proportion of BMAL1^+^ osteoblasts to total osteoblasts. *n* = 5 per group. The data are presented as the mean ± SD. For panels **a**–**f**, **h**, **i**: Two-way ANOVA combined with Tukey post hoc test was used to test the differences among all groups. For panel (**k**, **l**): unpaired, two-tailed Student’s *t* test was used to test the differences between OVX and OVX + Fx + vehicle groups; two-way ANOVA combined with Tukey post hoc test was used to test the differences between OVX + Fx, OVX + Fx mice treated with Pro, PTH, PTH + Pro groups. *P < 0.05, **P < 0.01, ***P < 0.001

Both plasma PTH and NE levels exhibit diurnal rhythms.^22,23^ It has been reported that βAR signals in osteoblasts regulate bone anabolic metabolism via circadian clock genes.^18^ Therefore, we further examined the expression of core clock genes (*Bmal1, Clock, Per1, Per2, Cry1 and Cry2*) (Fig. 5a–f) and found that NE significantly inhibited iPTH-induced *Bmal1* expression, which was further reversed by propranolol intervention (Fig. 5a). Consistently, Western blot analyses further confirmed the inhibitory effect of NE on iPTH-induced Bmal1 and RUNX2 expression, which was reversed by propranolol intervention (Fig. 5g–i). Moreover, double-label immunofluorescence staining revealed that the expression of Bmal1 was significantly increased in OCN^+^ osteoblasts from osteoporotic fracture mice treated with PTH, and this effect was further augmented by the combination of propranolol, as evidenced by significantly increased number of BMAL1^+^ osteoblasts (Fig. 5j, k) as well as the higher proportion of BMAL1^+^ osteoblasts to total osteoblasts in the mice treated with PTH and propranolol (Fig. 5l). These findings suggest that NE inhibits iPTH-induced Bmal1 expression in osteoblast cell line via βAR signaling in vivo and in vitro.

### βAR signaling inhibits iPTH-induced osteoblast differentiation via Bmal1

**Fig. 6:**
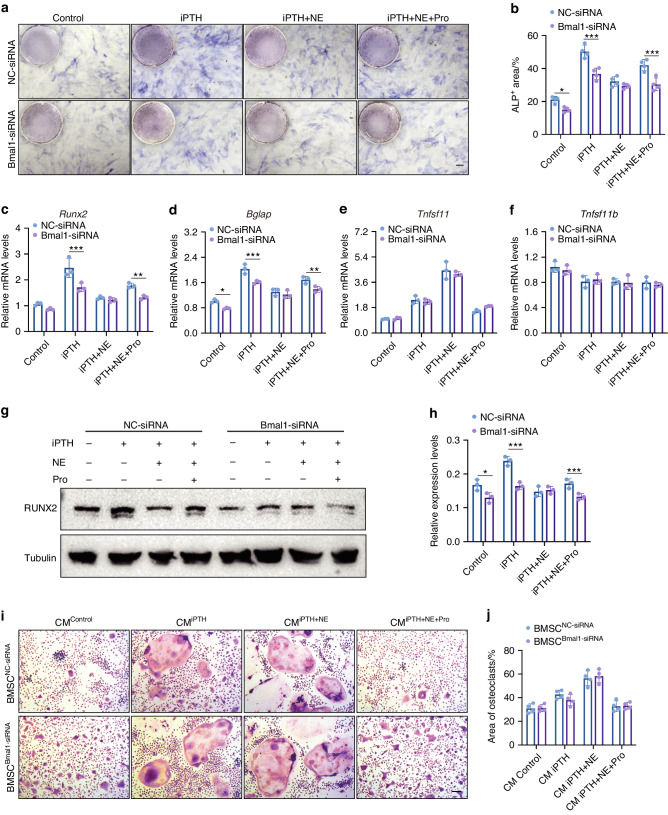
The effects of Bmal1 on iPTH-induced osteoblast differentiation and osteoblast-mediated osteoclastogenesis. ALP staining images (**a**) and quantification of the ALP positive areas (**b**) of negative control siRNA (NC-siRNA) or Bmal1-siRNA treated BMSCs further administrated with vehicle, iPTH, iPTH + NE or iPTH + NE + propranolol under osteogenic induction. Scale bar: 50 μm. *n* = 4 per group. **c**–**f** qRT-PCR analyses of *Runx2, Osteocalcin, Rankl and Opg* mRNA expression in BMSCs from different groups as indicated. Western blot images (**g**) and quantification of RUNX2 relative to tubulin (**h**) in BMSCs receiving different treatments. *n* = 3 per group. TRAP staining images (**i**) and quantification of TRAP^+^ osteoclast area (**j**) in BMMs treated with CM of BMSCs from different groups under osteoclastogenic induction. Scale bar: 50 μm. *n* = 4 per group. The data are presented as the mean ± SD. Two-way ANOVA combined with the Sidak post hoc test was used to test the differences between the NC-siRNA and Bmal1-siRNA groups in each treatment group

**Fig. 7:**
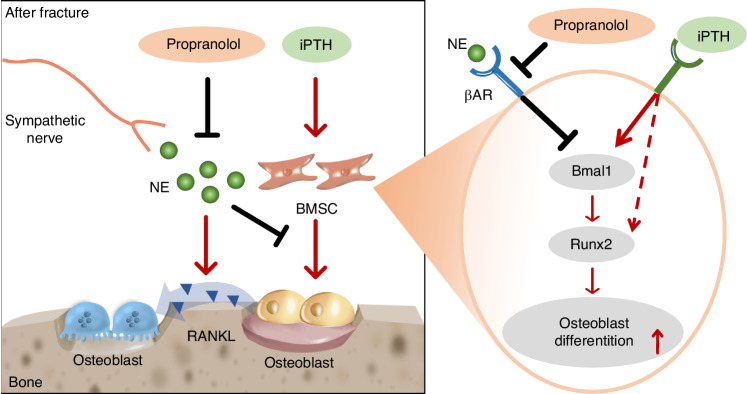
Schematic diagram showing the cellular and molecular mechanism by which propranolol augments the anabolic effect of PTH on systemic bone loss after fracture: The fracture triggers an increase in sympathetic tone. NE released by sympathetic nerves enhances osteoblast-mediated osteoclastic differentiation by promoting the secretion of RANKL in osteoblast cell lines. Conversely, NE inhibited PTH-induced osteoblast differentiation by inhibiting the expression of Bmal1 and Runx2. Propranolol enhances the osteogenic effect of PTH by inhibiting βAR signaling activated by NE

To further investigate whether propranolol enhances the osteogenic effect of PTH by regulating Bmal1, we downregulated Bmal1 in BMSCs using specific siRNAs, and the inhibitory efficiency of these siRNAs was confirmed by qRT‒PCR and Western blotting (Fig. S5). As shown by ALP staining (Fig. 6a, b), *Bmal1* knockdown partly but significantly inhibited the iPTH-induced osteoblast differentiation, suggesting that Bmal1 is involved in iPTH-induced osteogenic differentiation. Importantly, *Bmal1* knockdown blocked the rescue effect of propranolol on osteoblast differentiation in cells treated with iPTH + NE (Fig. 6a, b). qRT‒PCR and Western blot analyses further confirmed that Bmal1 knockdown suppressed iPTH− and iPTH + NE + Pro-induced *Runx2* and *Bglap* expression (Fig. 6c, d) as well as RUNX2 protein expression (Fig. 6g, h). However, *Bmal1* knockdown by siRNA had no notable effect on *Rankl* and *Opg* expression (Fig. 6e, f). Moreover, there was no obvious difference in osteoclastogenesis between BMMs treated with CM from BMSCs treated with NC-siRNA or Bmal1-siRNA in any of the groups (Fig. 6i, j), indicating that Bmal1 has no significant effect on osteoblast-mediated osteoclast differentiation. These results suggest that βAR signaling inhibits iPTH-induced osteoblast differentiation through Bmal1 and that propranolol blocks βAR signaling and increases Bmal1 expression, thereby promoting iPTH-induced osteoblast differentiation (Fig. 7).

## Discussion

Fractures are the most common and most serious complication of osteoporosis and can result in increased mortality and disability.^1^ The cumulative mortality rate for 12 months following an osteoporotic hip fracture was 33%.^24^ Recent fractures are considered one of the most reliable predictors of the risk of future fractures, with a higher risk within 2 years after fracture, which highlights the importance of timely treatment for systemic bone loss after fracture. This study revealed that fractures further accelerated bone loss in mice with OVX-induced osteoporosis. Consistent with our results, clinical research has shown that incident fractures are associated with an accelerated decrease in hip bone mineral density (BMD) in elderly women.^25^ Furthermore, our results showed that mice with fracture exhibit higher sympathetic tone, increased osteoclast formation and decreased osteoblast numbers. The β-receptor blocker propranolol significantly increased the trabecular bone mass in OVX mice after fracture, suggesting an important role for sympathetic tone in fracture-induced systemic bone loss.

PTH stimulates osteoblast differentiation and proliferation^26^ and indirectly activates osteoclasts by regulating RANKL and OPG in osteoblast cell lines.^27^ Differing from the efficacy of PTH observed in the treatment of postmenopausal osteoporosis, clinical studies involving the use of PTH after fracture have shown mixed results, ranging from positive effects to no effects on fracture healing.^28–30^ These findings suggest that fracture-induced changes in the internal environment may affect the effect of PTH on bone metabolism. Interestingly, we found that propranolol augmented the anabolic effects of PTH on systemic bone formation and fracture healing in mice with osteoporotic fractures. Importantly, we found that the activation of βAR signaling by NE blunted iPTH-induced osteoblast differentiation but enhanced RANKL expression in osteoblast lineage cells. Given the elevated sympathetic stress following fracture, the anabolic effect of PTH may be impaired in fracture subjects, which explains the potentiating effect of propranolol on PTH anabolism in mice with osteoporotic fractures. Consistent with our findings, Obri et al. provided evidence that sympathetic signaling favors PTH-stimulated Rankl expression in osteoblasts by increasing HDAC4 accumulation.^31^ These findings suggest that the combination of PTH and propranolol may be a better option for treating systemic bone loss after osteoporotic fractures and preventing recurrent fractures, which requires further clinical studies.

However, Hanyu et al. ^14^ reported that PTH showed no bone anabolic activity in global β2AR-deficient mice, demonstrating that β2AR signaling was indispensable for the anabolic action of PTH. These data seem to contrast with our results. However, propranolol works as a reversible antagonist that inhibits both β1AR and β2AR. Propranolol-mediated βAR inhibition differs from the permanent, irreversible and complete loss of function caused by β2AR gene knockout.

However, PTH exerts its anabolic effects through a variety of cell types. In addition to osteoblasts, osteocytes are crucial target cells for the anabolic action of PTH in bone.^32^ PTH regulates the expression of several osteocytic genes, including RANKL, sclerostin, DKK1 (Dickkopf Wnt Signaling Pathway Inhibitor 1), and MMP14 (matrix metalloproteinase), thereby controlling both bone formation and resorption.^32^ Additionally, periosteal stem cells, which significantly contribute to bone formation and fracture repair,^33^ are also responsive to PTH and play a role in the generation of fracture calluses.^34,35^ Therefore, it’s important to note that there may be other cell types that also contribute to the effects of PTH and propranolol on bone metabolism, and further research is needed to fully understand the complex interplay of these cellular mechanisms.

The circadian rhythm plays a crucial role in regulating various physiological processes, including hormone production, autonomic nervous system activity, cardiovascular rhythm and bone metabolism.^36–39^ Previous studies have shown that delayed bedtime and abnormal sleep duration are linked to low bone mineral density (BMD).^40^ Swanson CM et al. reported that three weeks of circadian disruption and sleep restriction resulted in a significant decrease in bone formation markers and an increase in sclerostin levels among adult men.^41^ Here, we found that PTH affects the expression of circadian clock genes in vivo and in vitro, and that knockdown of the circadian clock gene Bmal1 impairs the positive effect of iPTH on osteoblast differentiation. Interestingly, a clinical study of patients with postmenopausal osteoporosis reported that 12-month morning administration of teriparatide resulted in a greater increase in the lumbar spine BMD than evening administration.^42^ These findings suggest that circadian clock genes are involved in the regulation of osteogenic metabolism by PTH.

Circadian rhythms are driven by internal biological clock genes. BMAL1 is a core component of the circadian clock loop and participates in multiple biological processes during the development and remodeling of bone.^43^ Bmal1-deficient mice exhibit a low bone mass phenotype and a reduced number of osteoblasts.^44^ Furthermore, clock genes mediate the sympathetic regulation of osteoblastic bone formation.^45^ In this study, we demonstrated that NE-activated βAR signaling suppressed the PTH-induced increase in the expression of Bmal1 and Runx2, which was alleviated by propranolol; moreover, Bmal1 knockdown blocked the positive effects of PTH and propranolol on the differentiation of BMSCs into osteoblasts, indicating that NE interferes with the effect of PTH on osteogenesis by regulating Bmal1. These findings reveal a novel mechanism by which sympathetic stress modulates PTH-induced osteogenic differentiation and provide a potential pharmacological target for systemic bone loss after fracture.

In summary, we demonstrated that propranolol enhanced the anabolic effect of PTH in mice with osteoporotic fractures by increasing osteoblastic bone formation and reducing osteoclastic resorption. Mechanistically, NE impaired PTH-induced bone anabolism by inhibiting PTH-induced Bmal1 and Runx2 expression and promoting PTH-induced RANKL expression in osteoblast lineage cells, which was prevented by propranolol. Our study suggested that the combination of PTH and propranolol may offer new possibilities for effectively treating systemic bone loss and reducing the risk of subsequent fractures in patients with osteoporosis.

## Materials and methods

### Animals and treatments

All the experiments were approved by the Ethics Committee of Biomedical Science of Peking University (approval no. LA20210509). Twelve-week-old female C57BL/6 mice were used in this study. The mice were anesthetized and subjected to bilateral ovariectomy (OVX) as described previously.^46^ Two months later, the mice were further subjected to tibia fracture or sham operation as previous description with modification.^47^ Briefly, the mice were anesthetized with 2%-4% isoflurane gas and a syringe needle was inserted into the tibia canal for fixation. Fractures were created at the mid-diaphysis using scissors. X-ray confirmed the position of the intramedullary pin and fracture. OVX mice with fractures were further randomly assigned to four treatment groups: iPTH (rhPTH1-34; Shenzhen Salubris Pharmaceuticals Co, China), propranolol (MedChemExpress, New Jersey, USA), iPTH + propranolol, or vehicle. PTH was intermittently administrated by subcutaneous injection at 80 μg/kg once a day at approximately 10 am. Propranolol hydrochloride was dissolved in drinking water (0.5 g/L) and was delivered daily (changed once per 3 days). Mice were euthanized after 4 weeks of treatment. Tissues, including tibias, femurs, lumbar vertebrae and serum, were collected for further analyses.

### μCT

The bone samples were fixed in 4% paraformaldehyde for 24 h prior to scanning using a micro-CT scanner (Siemens, Erlangen, Germany). The scanner was set at a voltage of 80 kV, a current of 500 μA, a resolution of 8.82 μm per pixel, and an exposure time of 1 500 ms. To determine the bone mass and microstructure, the trabecular bone in the L5 vertebra beginning from the cranial growth plate and extending to the caudal growth plate was analyzed. For the femur analysis, the region of interest started from 0.15 mm proximal to the distal epiphyseal growth plate and was extended by 0.5 mm. To analyze callus formation, 100 continuous slices (50 slices up and 50 slices down from the fracture line) covering the middle of the newly formed callus were selected as the region of interest (ROI). Micro-CT software was used to calculate the bone volume (BV) and bone volume fraction (BV/TV) of the callus, excluding the native cortical bone.

### Biomechanics

A three-point bending test was performed to determine the mechanical properties of the fractured tibiae using a mechanical testing system (Landmark, MTS, Inc., Eden Prairie, MN, USA). The loading point is located in the middle of the tibiae, with two pivots spaced 8 mm apart. Each sample is loaded at 6 mm/min until the breaking point was reached. The ultimate strength was calculated from the load-deformation curve.

### Calcein double labeling

To assess dynamic bone formation, mice in different groups received intraperitoneal injections of 0.1% calcein solution (10 mg/kg body weight; Sigma) in PBS at 10 and 3 days before euthanasia. After euthanasia, the femurs were collected and fixed with 4% paraformaldehyde for 24 h, dehydrated in graded ethanol and embedded in methyl methacrylate. Using a hard sectioning microtome (EXAKT Cutting & Grinding System; EXAKT Advanced Technologies, Norderstedt, Germany), 50-μm-thick bone slices were obtained. Calcein double labeling was examined under a fluorescence microscope (Nikon). ImageJ v1.52 software was used to quantify the mineral apposition rate (MAR) of the trabecular bone.

### Histological and immunohistochemical analyses

The femora were decalcified using a 10% EDTA solution with constant shaking for a period of 21 days. The samples were subsequently dehydrated using graded ethanol, embedded in paraffin and cut into 5-μm-thick sections. Immunostaining was conducted using a standard protocol, in which the sections were incubated overnight at 4 °C with an anti-osteocalcin (OCN) antibody (1:100; Abcam; Britain). The primary antibody was detected using a Rabbit-specific HRP/DAB Detection IHC Kit (Abcam; UK), followed by counterstaining with hematoxylin. TRAP staining was performed using a commercial kit (Sigma-Aldrich; USA) in accordance with the manufacturer’s recommendations. The nuclei were stained using methyl green, and TRAP^+^ cells (red) were identified as osteoclasts. The OCN^+^ and TRAP^+^ areas on the trabecular bone surface were calculated in three sections per mouse using ImageJ v1.52 software.

### Immunofluorescence

OCN and Bmal1 double immunofluorescence staining was performed to evaluate the expression of BMAL1 in osteoblasts. Briefly, decalcified femora were dehydrated in 30% sucrose, embedded in OCT compound and cut into 5-μm-thick sections. After blocking with 5% donkey serum at room temperature for 30 min, the samples were incubated with an anti-BMAL1 antibody (1:100; Abcam; Britain) and an anti-OCN antibody (1:100; Abcam; Britain) overnight at 4 °C. Double-immunofluorescence staining was performed with a Treble-Fluorescence Immunohistochemical Mouse/Rabbit kit (Immunoway, USA) according to the manufacturer’s protocol. The number of BMAL1^+^ osteoblasts on the trabecular bone surface was calculated in three sections per mouse using ImageJ v1.52 software. The proportion of BMAL^+^ osteoblasts to total osteoblasts was also calculated.

### ELISA

We used commercial ELISA kits from Elabscience (Wuhan, China) to measure the serum OCN, CTX-I and NE levels following the manufacturer’s instructions. We read the optical density of each well at 450 nm using a microplate reader (Bio-Rad 680, Hercules, USA) and calculated the protein concentration for each sample based on the standard curve.

### BMSC culture

We isolated primary BMSCs from the femurs and tibias of C57BL/6 mice as described previously.^48^ Briefly, the femurs and tibias were extracted under sterile conditions and immersed in α-MEM (HyClone, Logan, USA) supplemented with 1% penicillin-streptomycin (PS; Beyotime, Shanghai, China). The bone marrow was flushed with α-MEM + 1% PS. The cells were washed and cultured in α-MEM supplemented with 10% fetal bovine serum (FBS) and 1% PS. Non-adherent cells were removed after 24 h of culturing with fresh complete α-MEM medium while the adherent cells were further cultured until they reached 90% confluence and passaged as needed.

### Osteogenic differentiation

BMSCs and BMSCs transfected with Bmal1-siRNA or NC-siRNA were plated in 24-well plates until 80% confluence. The BMSCs were then incubated with osteogenic differentiation medium, and treated under the following conditions: intermittent PTH (iPTH;100 ng/mL), iPTH + NE (10 μmol/L), iPTH (100 ng/mL) + NE (10 μmol/L) + propranolol (1 μmol/L) or vehicle (PBS). The iPTH treatment was conducted as previously described.^49^ Briefly, during the 48-hour incubation cycle, BMSCs were treated with PTH for the initial 6 hours, followed by 42 h of culture without PTH. The osteogenic differentiation medium was changed every 48 h. The conditioned media (CM) from the different groups were harvested and centrifuged at 2 000 × *g* for 10 min to collect the supernatant, which was used for subsequent experiments. After 7–14 days of differentiation culture, the cells were stained or harvested for total RNA and protein extraction for further analysis.

### ALP and ARS staining

After 7 days of osteogenic induction, the differentiated BMSCs were washed with PBS, fixed with 4% paraformaldehyde for 10 min, and stained for alkaline phosphatase (ALP) with a commercially available ALP staining kit (Solarbio, Beijing, China). Alizarin red S (ARS) staining was carried out after 14 days of differentiation, after which the cells were washed with PBS, fixed with 4% paraformaldehyde for 10 min, and then incubated with 2% ARS solution (Solarbio, Beijing, China) according to the manufacturer’s instructions. After washing with PBS, the stained cells were examined using an inverted microscope (Leica DMI6000B, Solms, Germany). The fractions of ALP^+^ and ARS^+^ areas were calculated using ImageJ v1.52 software.

### qRT‒PCR

Total cellular RNA was extracted with TRIzol Reagent (Invitrogen, USA) and cDNA was synthesized via reverse transcription of 1 μg of the total RNA using a commercial kit (Qiagen, Dusseldorf, Germany). qRT**‒**PCR (20 μL) was conducted on an ABI PRISM® 7900HT System (Applied Biosystems, USA). Relative mRNA levels were calculated by the comparative Ct (2^–ΔΔCT^) method using *Gapdh* for normalization. The sequences of primers used are listed in Table S1.

### Western blot

Total protein samples were separated via sodium dodecyl sulfate‒polyacrylamide gel electrophoresis (SDS–PAGE) and transferred to polyvinylidene fluoride (PVDF) membranes (Millipore, USA). After blocking with 5% nonfat milk for 1 h at room temperature, the membranes were incubated overnight at 4 °C with the appropriate primary antibodies, which included BMAL1 (1:1 000, Abcam, Cambridge, Britain), RUNX2 (1:500; ProteinTech, Chicago, USA), and tubulin (1:1 000; Affinity, Liyang, China). Then, the membranes were incubated for 1 h at room temperature with the appropriate secondary antibodies (1:5 000; Cell Signaling Technology, Danvers, USA). The blots were then detected with an enhanced chemiluminescence kit (Thermo Fisher Scientific).

### Transfection of siRNA

Four *Bmal1* siRNAs (Bmal1-siRNA #1, 2, 3 and 4) were synthesized by GenePharma (Shanghai, China). Cells transfected with negative control siRNAs (NC-siRNAs) were used as controls. The siRNAs were transfected into BMSCs using Lipofectamine® 3000 (Invitrogen, USA) according to the manufacturer’s instructions. The transfection efficiency was determined by qRT‒PCR and Western blot analysis. All siRNA sequences are listed in Table S2.

### BMM culture

BMMs were isolated from the long bones of 6-week-old C57BL/6 mice as described previously.^46^ Briefly, the bone marrow cells were flushed out using an injector and then cultured in α-MEM supplemented with 10% fetal bovine serum (FBS) (Gibco, USA), 100 U/mL penicillin and 100 μg/mL streptomycin (Solarbio, China), 30 ng/mL recombinant murine macrophage colony stimulating factor (M-CSF; PeproTech, USA). After 18 hours, we discarded the adherent cells and collected the floating cells that were subsequently cultured in a new flask to obtain the macrophages.

### Osteoclastogenic induction

The BMMs were cultured in 48-well plates in α-MEM (HyClone, Logan, USA) supplemented with 50 ng/mL of receptor activator for nuclear factor κB ligand (RANKL; Novoprotein, Shanghai, China) in the presence of vehicle (PBS), PTH (100 ng/mL), PTH + NE (10 μmol/L), PTH (100 ng/mL) + NE (10 μmol/L) + propranolol (1 μmol/L) or conditioned media from BMSCs treated with vehicle iPTH, iPTH + NE or iPTH + NE + propranolol as described above. Osteoclast formation was identified using a commercial TRAP kit (Sigma‒Aldrich, USA) according to the manufacturer’s instructions. The areas of TRAP^+^ (red) osteoclasts were measured by ImageJ 1.8.0 software.

### Statistical analysis

The data are presented as the mean ± SD. The unpaired, two-tailed Student’s *t* test was used for comparing mean differences between two groups. Two-way ANOVA combined with Turkey’s or Sidak post hoc test was used for multiple-group comparisons, as detailed in each figure legend. A difference with *P* < 0.05 was considered to indicate statistical significant. GraphPad Prism 9 software was used for statistical analyses.

### Supplementary information

Revised Supplemental material
