# Supplementary material for: β-Receptor blocker enhances the anabolic effect of PTH after osteoporotic fracture
Source: Bone Res. 2024 Mar 21;12:18. doi: 10.1038/s41413-024-00321-z (PMC10958005; doi:10.1038/s41413-024-00321-z)
Supplement: Supplementary file 1 — Revised Supplemental material [file 41413_2024_321_MOESM1_ESM.docx]

**Supplemental materials**

**β-Recept****or blocker enhances the anabolic effect of PTH after osteoporotic fracture**

Jie Huang^1^, Tong Wu^1^, Yi-Rong Jiang^1^, Xuan-Qi Zheng^1^, Huan Wang^1^, Hao Liu^1^, Hong Wang^1,2,3^, Hui-Jie Leng^1,2,3^, Dong-Wei Fan^1,2,3^, Wan-Qiong Yuan^1,2,3^, Chun-Li Song^1,2,3^*

1. Department of Orthopaedics, Peking University Third Hospital 100191, Beijing, China

2. Beijing Key Laboratory of Spinal Disease, Beijing 100191, China

3. Engineering Research Center of Bone and Joint Precision Medicine 100191, Beijing, China

Correspondence: Chun-Li Song (schl@bjmu.edu.cn)

These authors contributed equally: Jie Huang, Tong Wu

**
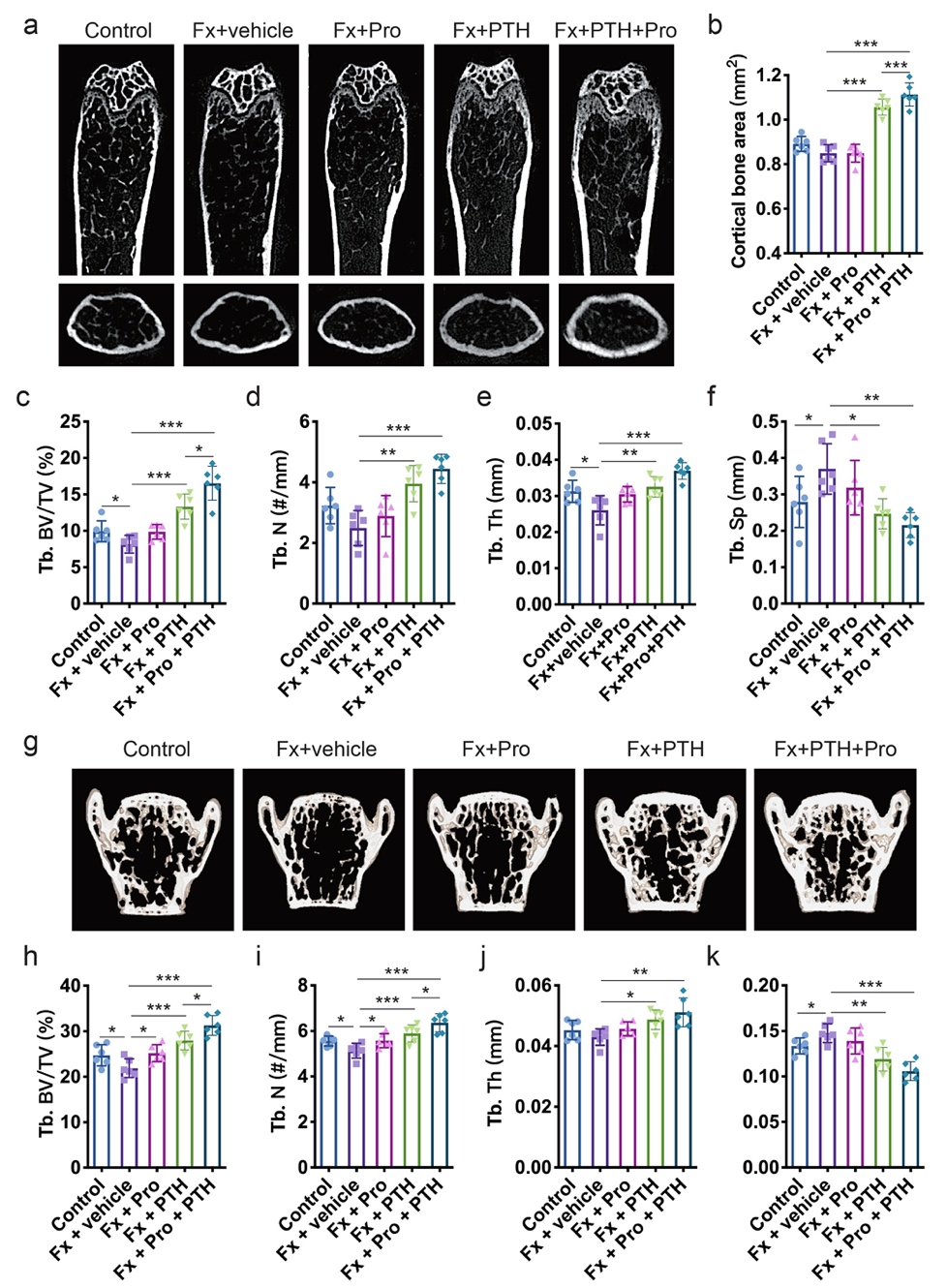
**

**Fig. S1** Propranolol increases PTH-induced bone formation of femurs and L5 in mice after fracture. **a** Representative μCT images of femurs from Control, Fx (fracture) mice treated with Pro (propranolol), PTH, PTH + Pro, or vehicle groups. **b-f** Quantitative μCT analysis of the cortical bone area (**b)**, trabecular bone volume fraction (Tb. BV/TV) (**c**), trabecular bone number (Tb. N) (**d**), trabecular bone thickness (Tb. Th) (**e**), trabecular bone separation (Tb. Sp) (**f**); n=6 per group. **g** Representative μCT images of L5. **h-k** Quantitative μCT analysis of trabecular bone microarchitecture of L5; n=8 per group. The data are presented as the mean ± SD. Unpaired, two-tailed Student’s t-test was used to test the differences between the Control and Fx + vehicle groups; two-way ANOVA combined with Tukey’s post hoc test was used to test the differences between Fx mice treated with Pro, PTH, PTH + Pro or vehicle groups. **P* < 0.05, ***P* < 0.01, ****P* < 0.001.

**
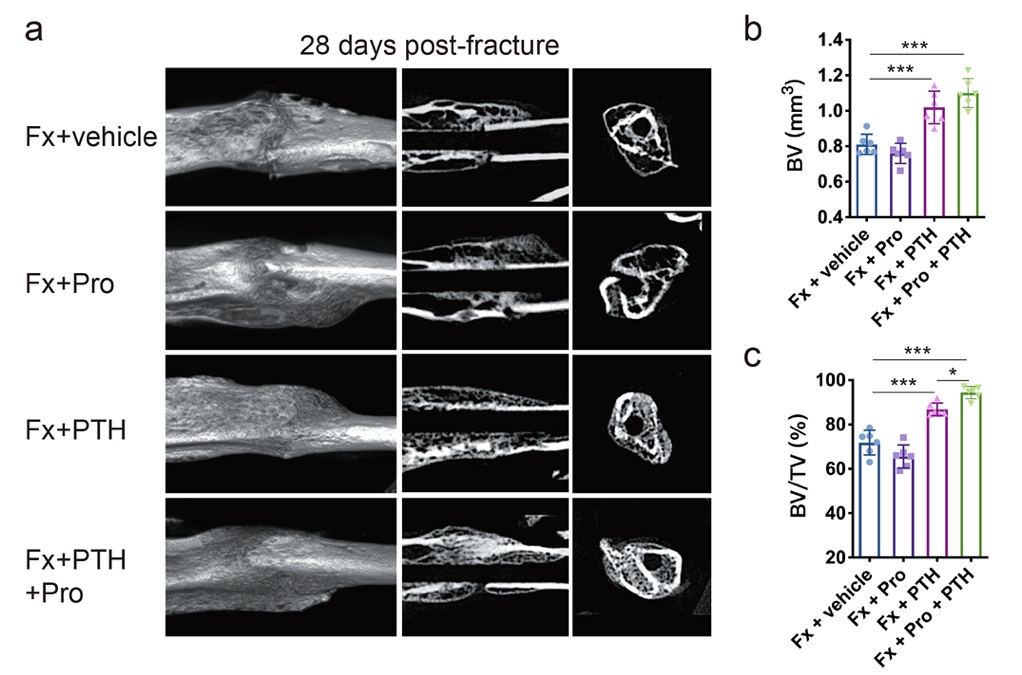
**

**Fig. S2** Propranolol facilitates the effect of PTH on fracture healing. **a** μCT 3D reconstructions, axial cross-sectional and coronal cross-sectional images of fractured tibias from mice treated with PTH, Pro, PTH + Pro or vehicle on day 28 after fracture. **b, c** Quantitative analysis of the bone volume (BV) and the bone volume fraction (BV/TV) of calluses. n=6 per group. The aata are presented as the mean ± SD. Two-way ANOVA combined with Tukey’s post hoc test was used to test the differences among all groups. **P* < 0.05, ***P* < 0.01, ****P* < 0.001.

**
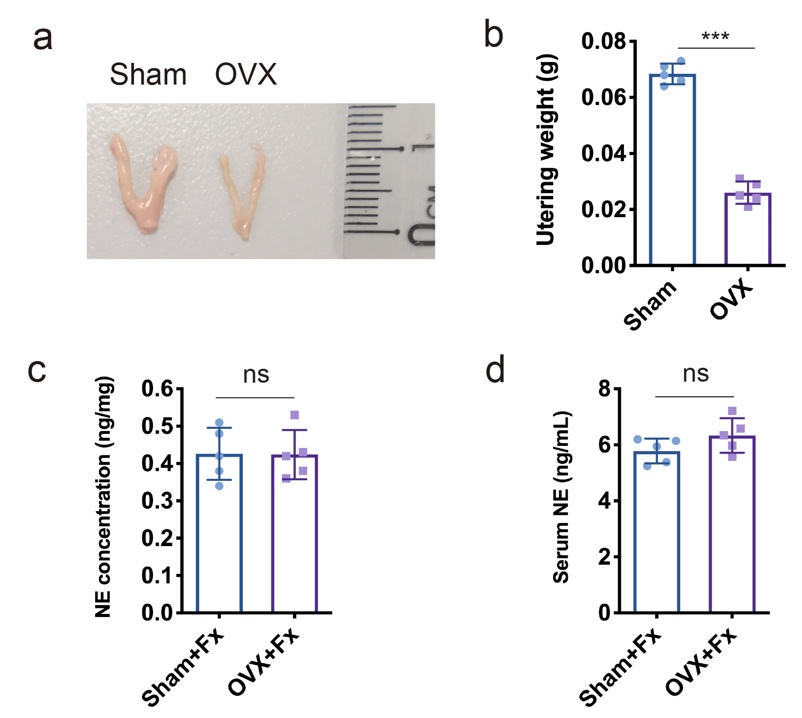
**

**Fig. S3** NE levels of fracture sites and serum in OVX and sham mice. **a** Representative images showing uterine morphology from sham and OVX groups. **b** Uterine weight in different groups. n = 5 per group. **c** NE concentration in fracture sites from sham and OVX groups. n = 5 per group. **d** serum NE concentrations of sham and OVX groups. n = 5 per group. The data are presented as the mean ± SD. Unpaired, two-tailed Student’s t-test was used to test the differences between sham and OVX groups. **P* < 0.05, ***P* < 0.01, ****P* < 0.001.

**
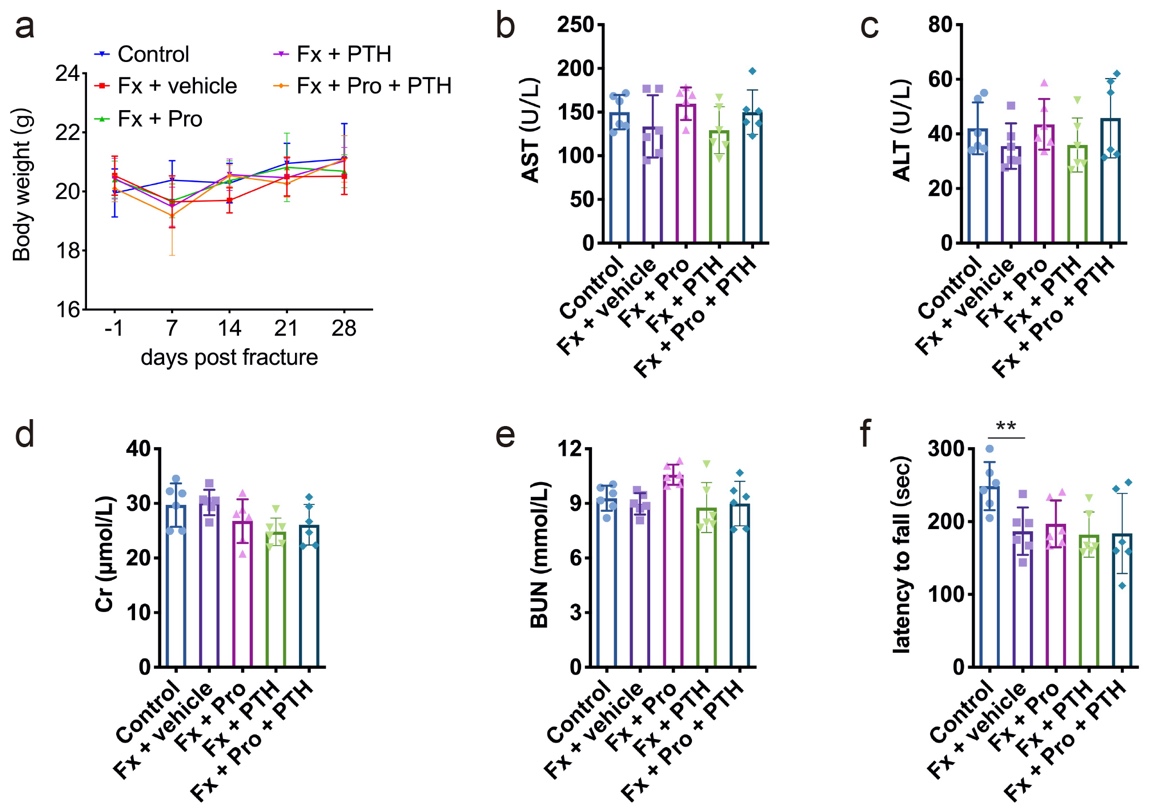
**

**Fig. S4** Effects of PTH and propranolol on body weight, liver and kidney function and fatigue in fractured mice. **a** The body weight record of control group and mice treated with propranolol, PTH, PTH + propranolol, or vehicle groups within 4 weeks of fracture. n = 6 per group. **b-e** Serum AST (aspartate transaminase), ALP (alanine transaminase), Cr (creatinine), and BUN (blood urea nitrogen) levels of different treatment groups. n = 6 per group. **f** Rotarod scores of each group on the rotarod test at day 28 after fracture. n=6 per group. The data are presented as the mean ± SD. Unpaired, two-tailed Student’s t-test was used to test the differences between Control and Fx + vehicle groups; two-way ANOVA combined with Tukey’s post hoc test was used to test the differences between Fx mice treated with Pro, PTH, PTH + Pro or vehicle groups. **P* < 0.05, ***P* < 0.01, ****P* < 0.001.

**
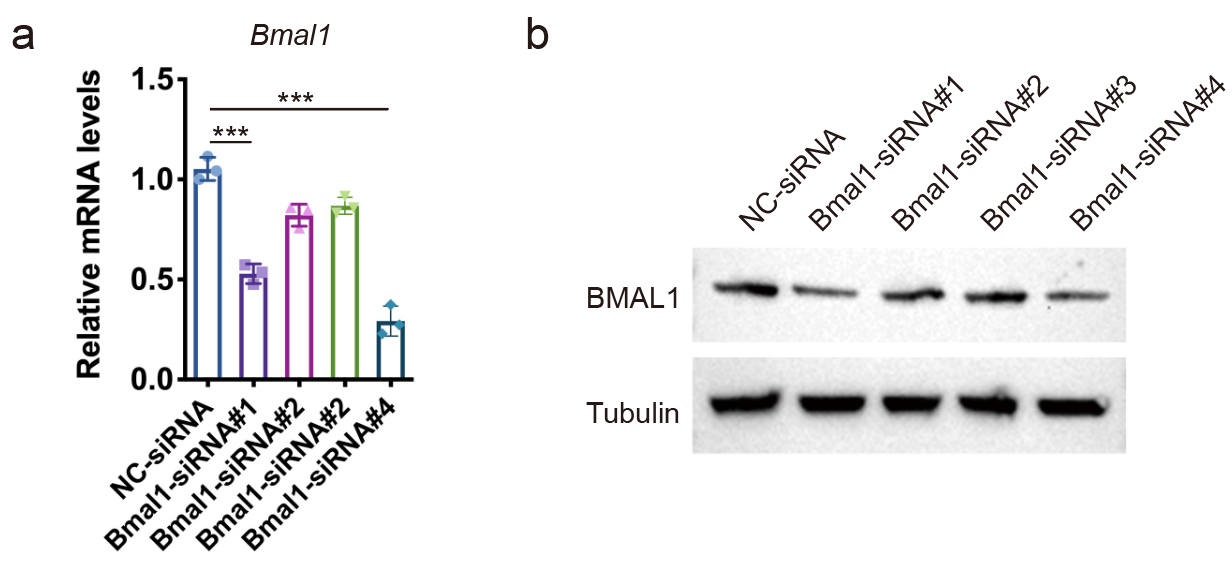
**

**Fig. S5** Inhibitory efficiency of Bmal1-siRNAs transfection. **a** qRT-PCR analysis of *Bmal1* expression in BMSCs transfected with the *Bmal1*-targeting siRNAs (siNPY) or the negative control siRNAs (NC-siRNA) for 24 h. n = 3 per group. **b** Western blot images of BMAL1 in BMSCs receiving siRNAs transfection. Bmal1-siRNA#4 was used in subsequent transfection experiments due to its higher knockdown efficiency. The data are presented as the mean ± SD. One-way ANOVA combined with Dunnett post hoc test was used to test the differences between the control group (NC-siRNA) and the other groups (Bmal1-siRNA#1-4). **P* < 0.05, ***P* < 0.01, ****P* < 0.001.

**Table S1. Primers for qRT-PCR**

| **Gene** | **Primer (5’→3′)** |
| --- | --- |
| *Alpl* | Forward: 5′- CCAACTCTTTTGTGCCAGAGA-3′ |
|  | Reverse: 5′-GGCTACATTGGTGTTGAGCTTTT-3′ |
| *Bglap* | Forward: 5′-TTCTGCTCACTCTGCTGACC-3′ |
|  | Reverse: 5′- TTAAGCTCACACTGCTCCCG-3′ |
| *Runx2* | Forward: 5′- GACTGTGGTTACCGTCATGGC-3′ |
|  | Reverse: 5′- CGTGGTACCAAGAGGACAGAGT-3′ |
| *Tnfsf11* | Forward: 5′-AGCCATTTGCACACCTCAC-3′ |
|  | Reverse: 5′- CGTGGTACCAAGAGGACAGAGT-3′ |
| *Tnfsf11b* | Forward: 5′- GTTTCCCGAGGACCACAAT-3′ |
|  | Reverse: 5′- CCATTCAATGATGTCCAGGAG-3′ |
| *Bmal1* | Forward: 5′- TGACCCTCATGGAAGGTTAGAA-3′ |
|  | Reverse: 5′-GGACATTGCATTGCATGTTGG-3′ |
| *Per1* | Forward: 5′-TGAAGCAAGACCGGGAGAG-3′ |
|  | Reverse: 5′- CACACACGCCGTCACATCA-3′ |
| *Per2* | Forward: 5′-TGAAGCAAGACCGGGAGAG-3′ |
|  | Reverse: 5′- AACTCGCACTTCCTTTTCAGG-3′ |
| *Cry1* | Forward: 5′- CACTGGTTCCGAAAGGGACTC-3’ |
|  | Reverse: 5′-CTGAAGCAAAAATCGCCACCT- 3′ |
| Cry2 | Forward: 5′- CACTGGTTCCGCAAAGGACTA -3’ |
|  | Reverse: 5′- CCACGGGTCGAGGATGTAGA- 3′ |
| *Clock* | Forward: 5′- AGAACTTGGCATTGAAGAGTCTC-3′ |
|  | Reverse: 5′- GTCAGACCCAGAATCTTGGCT-3′ |
| *Gapdh* | Forward: 5′- AGGTCGGTGTGAACGGATTTG-3′ |
|  | Reverse: 5′-TGTAGACCATGTAGTTGAGGTCA-3′ |

**Table S2. siRNA sequences**

| **siRNA** | **Sequences (5’→3′)** |
| --- | --- |
| Negative control | Sense: UUCUCCGAACGUGUCACGUTT |
|  | Antisense: ACGUGACACGUUCGGAGAATT |
| Bmal1#1 | Sense: GCAAACUACAAGCCAACAUTT |
|  | Antisense: AUGUUGGCUUGUAGUUUGCTT |
| Bmal1#2 | Sense: GCAACAGGCCUUCAGUAAATT |
|  | Antisense: UUUACUGAAGGCCUGUUGCTT |
| Bmal1#3 | Sense: CCUCAAUUAUAGCCAGAAUTT |
|  | Antisense: AUUCUGGCUAUAAUUGAGGTT |
| Bmal1#4 | Sense: GCGGAGGAAAUCAUGGAAATT |
|  | Antisense: UUUCCAUGAUUUCCUCCGCTT |

**Methods**

**Animals and treatments**

Twelve-week-old female C57BL/6 mice were used. The mice were subjected to tibia fracture or sham operation as previous description. Fractured mice further randomly assigned to four treatment groups: iPTH (rhPTH1-34; Shenzhen Salubris Pharmaceuticals Co, China), propranolol (MedChemExpress, New Jersey, USA), iPTH + propranolol, or vehicle respectively. Sham-operated mice were served as control. PTH was intermittently administrated by subcutaneous injection at 80μg/kg once a day around 10 am. Propranolol hydrochloride was dissolved in drinking water (0.5 g/L) and was delivered daily (changed once per 3 days). The mice were tested for body weight once a week for 4 weeks. Then, the mice were euthanized after 4 weeks of treatment. Tissues, including tibias, femurs, lumbar vertebrae and serum, were collected for further analyses.

To compare the NE levels of OVX mice and normal mice after fracture, twelve-week-old female C57BL/6 mice were generally anesthetized and were subjected to bilateral ovariectomy (OVX) or a sham operation as described previously. A month later, the mice were subjected to tibia fracture. The mice were euthanized one week following the fracture, and the uteruses were subsequently weighed to assess the effectiveness of the OVX operation. Additionally, the fractured tibias and serum samples were collected to determine the levels of norepinephrine (NE) via ELISA.

**Rotarod test**

The Rotarod test was used to detect fatigue levels in the mice after 4 weeks of intervention. the mice were trained for three days prior to the test. On the fourth day, the mice were placed on an accelerating rotarod cylinder, and their latency time was measured as the speed increased from 5 to 40 rpm over 5 minutes. A trial ended if the animal fell off the rungs or gripped the device and spun around for 2 consecutive revolutions without attempting to walk on the rungs. The motor test data are presented as the mean latency time (averaged over three trials) on the rotarod.

**Liver and kidney function tests**

The liver and kidney function of fractured mice were evaluated after 4 weeks of intervention. The levels of serum aspartate transaminase (AST), alanine transaminase (ALP), creatinine (Cr), and blood urea nitrogen (BUN) were measured using commercial kits from Rayto (Shenzhen, China) and tested by automatic blood biochemical analysis instruments (Rayto, Shenzhen, China).
